# Non-Coding RNAs in Vascular Calcification: Regulatory Networks, Functional Diversity, and Therapeutic Insights

**DOI:** 10.3390/cells15151337

**Published:** 2026-07-25

**Authors:** Yingkun Sheng, Ziyan Yin, Zile Zhang, Jingyi Shi, Xiao Wang, Jian Zhang, Xiaoxiao Hu, Weiling Hong

**Affiliations:** 1 Xingzhi College, Zhejiang Normal University, Jinhua 321100, China; yingkunsheng@zjnu.edu.cn (Y.S.); 15757685765@163.com (Z.Y.); 13738633239@163.com (Z.Z.); 2 Jinhua Advanced Research Institute, Jinhua 321019, China; sjingyiirene@163.com (J.S.); wangxiao20190605@163.com (X.W.); 3 School of Engineering, Westlake University, Hangzhou 310024, China; zhangjian@westlake.edu.cn

**Keywords:** non-coding RNAs, vascular calcification, miRNAs, lncRNAs, signaling pathways

## Abstract

**Highlights:**

**What are the main findings?**

- This is the first systematic categorization of calcification-regulating miRNAs by signaling pathways, revealing crosstalk and lncRNA/circRNA heterogeneity.
- Non-coding RNAs show context-dependent functional duality, with the same molecule exerting opposite effects via different targets and microenvironments.

**What are the implications of the main findings?**

- The pathway network provides a theoretical foundation for designing multi-target combination therapies against calcification.
- The duality underscores the need for disease-selective delivery in RNA therapeutics and supports circulating ncRNAs as non-invasive biomarkers.

**Abstract:**

Vascular calcification is an ectopic calcium salt deposition process that lacks effective treatment under pathological conditions such as chronic kidney disease, diabetes, and aging. This review, for the first time, systematically categorizes miRNAs involved in the regulation of vascular calcification according to signaling pathways, clearly revealing the regulatory nodes and crosstalk among different pathways (Wnt/β catenin, BMP/Smad, NF κB, etc.), thereby providing a pathway-level theoretical basis for precision targeting. It also dissects the functional duality and microenvironment dependency of miRNAs—the same miRNA can exert opposite effects in different contexts, suggesting that miRNA-based drug development must focus on disease-selective targeted delivery to avoid the risk of functional reversal. This article further comprehensively summarizes the heterogeneous regulatory patterns of lncRNAs, the unique regulatory modes of circRNAs, and preliminary evidence suggesting the involvement of emerging non-coding RNAs (piRNAs, tsRNAs) and integrates the potential of circulating non-coding RNAs as non-invasive biomarkers and prospects for targeted therapeutic translation. This review provides a panoramic view of the network-based regulation of vascular calcification by non-coding RNAs, laying a theoretical foundation for precision diagnosis and multi-target combination intervention.

## 1. Introduction

Vascular calcification (VC) is an actively regulated pathophysiological event involving ectopic calcium phosphate deposition within the vessel wall—particularly the arterial intima and media—and in cardiac valves, contrasting with the earlier notion that it was merely a passive degenerative process [1]. Under pathological conditions such as chronic kidney disease (CKD), diabetes, aging, and atherosclerosis, vascular calcification significantly increases the risk of cardiovascular events and all-cause mortality in patients and has become a key clinical challenge in the management of cardiovascular diseases worldwide [2]. However, despite increasing insights into the pathogenesis of vascular calcification, there is currently no formally approved effective drug for treating vascular calcification [3]. Therefore, developing molecular mechanism-based targeted therapeutic strategies has become an important direction in the field of vascular calcification.

Vascular calcification encompasses several distinct disease entities that differ in their cellular origin and pathogenic mechanisms. Medial calcification primarily affects VSMCs in the arterial media and is commonly associated with CKD, diabetes, and aging [2]. Intimal/atherosclerotic calcification occurs within atherosclerotic plaques and involves VSMC phenotypic switching in concert with inflammatory cells [4]. Calcific aortic valve disease (CAVD) affects valvular interstitial cells (VICs) in the aortic valve leaflets and represents a distinct pathobiological process [5]. In this review, we explicitly distinguish between VSMC-mediated calcification and VIC-mediated valve calcification when discussing ncRNA functions, as these cell types differ in their differentiation state, microenvironment, and responses to ncRNA regulation. The discussion is organized by signaling pathways to highlight regulatory nodes and crosstalk, with cell type information provided in the summary tables.

To achieve molecular mechanism-based targeted therapy, it is first necessary to clarify the key signaling pathways driving vascular calcification and their cross-regulatory networks, which together form a complex network that synergistically drives the calcification process. Among these pathways, abnormal activation of the Wnt/β-catenin signaling pathway drives the osteogenic phenotypic transformation of vascular smooth muscle cells (VSMCs) and is considered a core event in calcification initiation [6]. The BMP/Smad osteogenic differentiation axis, through phosphorylation of Smad1/5/8, upregulates Runx2 and osteocalcin expression and plays a key role in CKD-related vascular calcification [7]. The NF-κB-mediated inflammatory response pathway is also deeply involved, promoting pyroptosis and osteogenic gene expression by activating IL-6/STAT3 and the NLRP3 inflammasome [8]. In addition, endoplasmic reticulum (ER) stress (the PERK/ATF4 branch) and autophagy pathway disturbances have gained increasing attention as emerging signaling nodes, forming positive crosstalk with the above pathways [9]. These signaling networks do not operate independently but synergistically exacerbate calcium salt deposition through multiple feedback loops, suggesting the necessity of multi-target combination intervention.

In recent years, non-coding RNAs (including miRNAs, lncRNAs, and circRNAs) have been shown to extensively regulate vascular calcification-related signaling pathways through mechanisms such as competing endogenous RNA (ceRNA) networks, exosome-mediated intercellular communication, and epigenetic modifications. For example, miR-133a and the miR-30 family inhibit osteogenic differentiation of VSMCs by directly targeting Runx2 or Smad3, exerting protective effects [10,11], whereas miR-21-5p promotes calcification in different disease contexts by targeting TGFBI, Crim1, or Tpm1, exhibiting functional duality [12,13,14]. Long non-coding RNAs such as MALAT1, NEAT1, and H19 significantly exacerbate valve and vascular calcification by sponging protective miRNAs or binding epigenetic regulators such as EZH2 [15,16]. Circular RNAs (e.g., circ-GALK2, circZBTB44) have recently been reported to participate in calcification regulation through peptide-encoding or sponging mechanisms, further expanding the functional dimensions of non-coding RNAs [17,18]. Circulating non-coding RNAs (e.g., miR-125b, miR-223, miR-211) show altered expression in patient sera and have been used as non-invasive biomarkers to assess calcification severity and prognosis [19,20,21]. These findings fully reveal the central role of non-coding RNAs in the regulatory network of vascular calcification, laying a solid foundation for the development of non-coding RNA-based diagnostic markers and targeted therapeutic strategies.

## 2. Regulatory Roles of miRNA in Vascular Calcification

### 2.1. miRNAs Targeting the Wnt/β-Catenin Signaling Pathway

**Figure 1:**
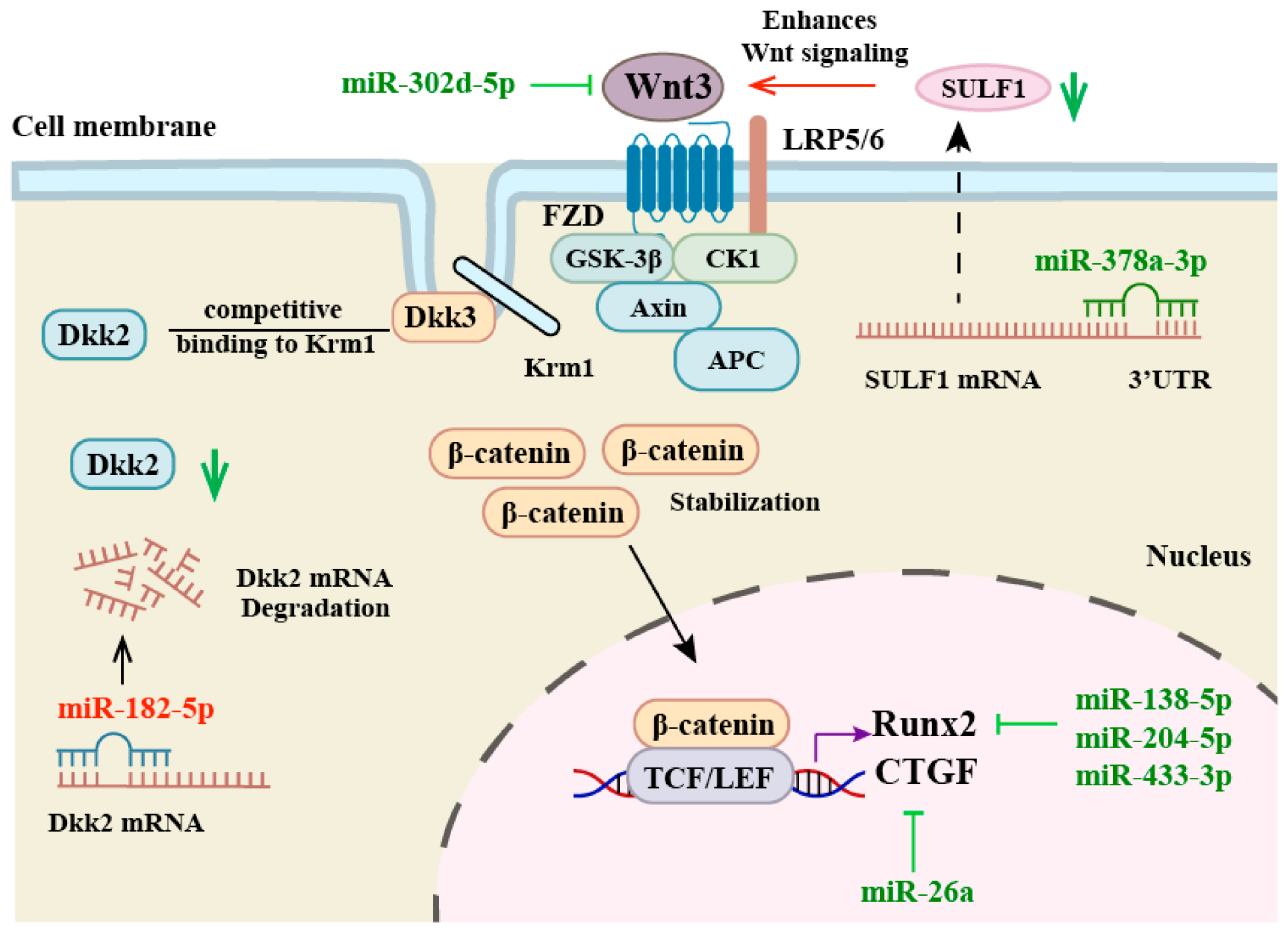
miRNAs targeting the Wnt/β-catenin signaling pathway. Upstream activating signals in this pathway include Wnt ligands (e.g., Wnt3, Wnt3a, Wnt7b), BMP-2, etc. Furthermore, pathway activity is negatively regulated by extracellular antagonist Dkk2 and modulated by molecules such as the sulfatase SULF1, which affects the distribution and signaling intensity of Wnt ligands by modifying heparan sulfate proteoglycans. When canonical Wnt signaling is activated, the activity of the cytoplasmic β-catenin degradation complex (GSK-3β, CK1, Axin, APC) is inhibited, allowing β-catenin to stabilize and translocate into the nucleus, where it directly initiates transcription of downstream target genes, especially Runx2, thereby inducing the expression of downstream effectors such as CTGF and driving VSMCs toward an osteoblast-like phenotype and calcium deposition. Red miRNAs indicate promotion of Wnt/β-catenin signaling, thereby exacerbating vascular calcification. Green miRNAs indicate inhibition of Wnt/β-catenin signaling, thereby retarding vascular calcification. Red arrows indicate activation of the signaling pathway, while green arrows indicate suppression. Black arrows indicate the direction of the signaling pathway.

In the regulatory network of the Wnt/β-catenin signaling pathway, multiple miRNAs participate in the regulation of vascular and valvular calcification by targeting molecules at different levels (Figure 1, Table S1). At the upstream ligand level, miR-302d-5p in endothelial cell-derived exosomes directly targets and inhibits Wnt3 ligand, thereby blocking the canonical Wnt/β-catenin signaling pathway and alleviating VSMC calcification and senescence [22]. At the extracellular regulatory molecule level, miR-182-5p promotes osteogenic differentiation of valvular interstitial cells (VICs) and progression of aortic valve calcification by targeting and inhibiting Dkk2, thereby relieving the negative regulation of the Wnt/β-catenin signaling pathway by the Krm1-Dkk2 complex [23]. As a protective factor, miR-378a-3p inhibits VSMC calcification by targeting and inhibiting SULF1, thereby blocking the Wnt/β-catenin signaling pathway; its expression is downregulated in the serum of CKD patients, leading to upregulation of SULF1 and thus promoting calcification [24]. At the downstream level of the signaling pathway, miR-26a inhibits VSMC calcification by targeting connective tissue growth factor (CTGF)—a key downstream effector of the Wnt/β-catenin pathway—and regulating the OPG/RANKL signaling pathway [25]. miR-138-5p directly targets RUNX2 and thereby blocks Wnt/β-catenin signaling, inhibiting osteogenic differentiation and calcium deposition of VICs [26]. Similarly, after miR-204-5p is delivered to VSMCs via exosomes from AT2 receptor agonist-stimulated macrophages, it alleviates phosphate-induced vascular calcification by targeting and inhibiting RUNX2 and blocking the Wnt/β-catenin pathway [27]. In addition, miR-433-3p, a protective factor against vascular calcification, is downregulated in the serum of patients with type 2 diabetes, thereby relieving its inhibition of the Wnt/β-catenin and RANKL/RANK/OPG signaling pathways and indirectly promoting calcification [28].

### 2.2. miRNAs Targeting the BMP/Smad/Runx2 Osteogenic Axis

#### 2.2.1. miRNAs Targeting Runx2

**Figure 2:**
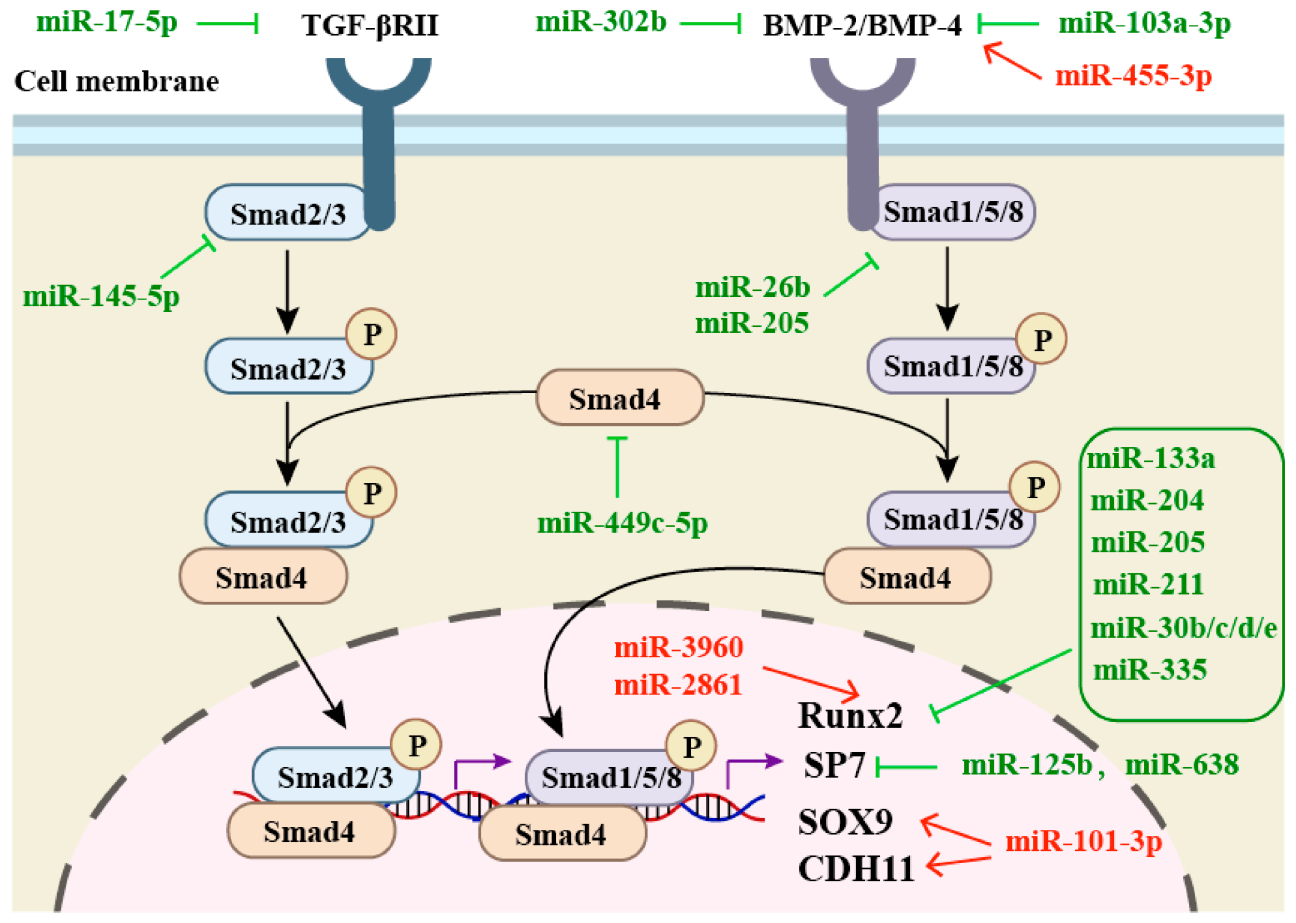
miRNAs targeting the BMP/Smad signaling pathway. The upstream-to-downstream hierarchy of the BMP/Smad signaling pathway in vascular and valvular calcification can be summarized as follows: the pathway begins with upstream receptors of the TGF-β/BMP superfamily (e.g., TGF-βRII); upon stimulation by ligands such as BMP2 and BMP4, the signal is sequentially transmitted to intracellular Smad molecules (including Smad1, Smad3, and the co-mediator Smad4), which then activate downstream osteogenic transcription factors (Runx2, Osterix/SP7, SOX9) as well as CDH11, a cell adhesion molecule positively regulated by BMP. Red miRNAs indicate promotion of vascular calcification; green miRNAs indicate inhibition of vascular calcification. Red arrows indicate activation of the signaling pathway, while green arrows indicate suppression. Black arrows indicate the direction of the signaling pathway.

In the regulation of vascular calcification and osteogenic differentiation, several miRNAs exert inhibitory effects by directly targeting the osteogenic transcription factor Runx2 (Figure 2, Table S1). Specifically, in high-phosphate-induced vascular calcification models, miR-211 expression is downregulated, and its direct inhibition of Runx2 is weakened, thus promoting osteogenic differentiation and calcification of VSMCs [29]. Downregulation of miR-204 may promote VSMC calcification via Runx2 [30], and in a high-phosphate environment, miR-204 itself is downregulated due to DNMT3a-mediated promoter hypermethylation; overexpression of miR-204 can reverse the calcification process by targeting Runx2, forming a miR-204/DNMT3a negative feedback loop [31]. Similarly, in a high-phosphate-induced model, miR-205 negatively regulates osteogenic-like differentiation by targeting Runx2 and Smad1 [32]. In a BMP2-induced VSMC calcification model, miR-133a expression is downregulated. Overexpression of miR-133a reduces calcification by downregulating Runx2 and osteocalcin levels, whereas inhibition of its expression exacerbates the calcification process [10]. Members of the miR-30 family also exert inhibitory effects by targeting Runx2. miR-30b and miR-30c directly bind Runx2 mRNA to inhibit its translation, and their levels are reduced in calcified human coronary arteries [11]. Additionally, miR-30 inhibits osteogenic differentiation of adipose-derived mesenchymal stem cells by suppressing Runx2 mRNA expression [33]. Under mechanical unloading conditions (e.g., prolonged bed rest or spaceflight), miR-30b/c/d/e expression is upregulated and inhibits osteoblast differentiation by suppressing Runx2 [34]. In aortic valve calcification, miR-204 expression is significantly downregulated, and its overexpression blocks TGF-β1-induced osteogenic reprogramming of VICs by targeting Runx2 and Osx [35]. Moreover, miR-335, delivered to VSMCs via bone marrow mesenchymal stem cell-derived exosomes, antagonizes osteogenic-like differentiation and calcification by targeting SP1 to downregulate RUNX2 [36]. Contrary to the above inhibitory miRNAs, miR-3960 and miR-2861 are upregulated during osteogenic transdifferentiation of VSMCs and increase Runx2 protein levels by targeting and inhibiting Hoxa2 and HDAC5, respectively. Runx2 transcriptionally activates this miRNA cluster, forming a positive feedback loop that promotes vascular calcification [37].

#### 2.2.2. miRNAs Targeting BMP/Smad or Related Molecules

Following the upstream-to-downstream order of the BMP/Smad signaling pathway, multiple miRNAs regulate vascular or valvular calcification by targeting different nodes of this pathway (Figure 2, Table S1). At the upstream level of the pathway, under high-glucose conditions, macrophage-derived extracellular vesicles deliver miR-17-5p, which targets and inhibits transforming growth factor-β receptor II (TGF-βRII, an upstream receptor of the TGF-β/BMP superfamily), thereby attenuating TGF-β1-induced osteogenic differentiation and calcium deposition of VSMCs [38]. At the BMP-2 signaling level, miR-302b is downregulated in a vascular calcification model of chronic renal failure rats; overexpression of miR-302b improves calcium-phosphate metabolic disorders by inhibiting the BMP-2/Runx2/Osterix signaling pathway, thus alleviating vascular calcification [39]. At the BMP4 signaling level, miR-103a-3p is downregulated in plasma extracellular vesicles from patients with aortic stenosis; overexpression of miR-103a-3p inhibits osteogenic differentiation and calcification of VICs by regulating the BMP4/RUNX2 signaling axis [40]. Conversely, miR-455-3p is upregulated in plasma extracellular vesicles from the same disease; inhibition of miR-455-3p alleviates osteogenic differentiation and calcification of VICs by regulating the BMP4/RUNX2 signaling axis [40]. At the Smad molecule level, miR-26b blocks fibroblast-derived BMP4-mediated pro-calcific communication by targeting and inhibiting Smad1 expression in VSMCs, and its loss drives spontaneous aortic microcalcification [41]. In addition, miR-145-5p prevents VSMC osteogenic differentiation by directly binding and inhibiting Smad3 expression [42]. miR-449c-5p is significantly downregulated in calcified human aortic valves; overexpression of miR-449c-5p directly targets and inhibits Smad4, thereby blocking the TGF-β/Smad signaling pathway and suppressing osteogenic differentiation and calcification of VICs [43]. At the level of the downstream osteogenic transcription factor Osterix (SP7), miR-125b negatively regulates osteogenic-like differentiation and calcification of VSMCs by targeting SP7 [44]. miR-638 is upregulated in calcified aortic valves; functional experiments confirm that it exerts an anti-osteogenic differentiation effect by targeting SP7, suggesting that its upregulation may be a compensatory protective response to calcification stress, but it is insufficient to reverse an already initiated calcification process [45]. Furthermore, miR-101-3p is significantly upregulated in calcified human aortic valves; overexpression of miR-101-3p promotes osteogenic differentiation and calcification of human VICs by directly targeting and inhibiting CDH11 (a cell adhesion molecule whose expression is positively regulated by BMP signaling) and SOX9 (a chondrogenic transcription factor downstream of BMP), while inhibition of miR-101-3p restores CDH11 and SOX9 levels and effectively prevents the calcification process [46].

### 2.3. miRNAs Targeting the NF-κB Inflammatory Signaling Pathway

**Figure 3:**
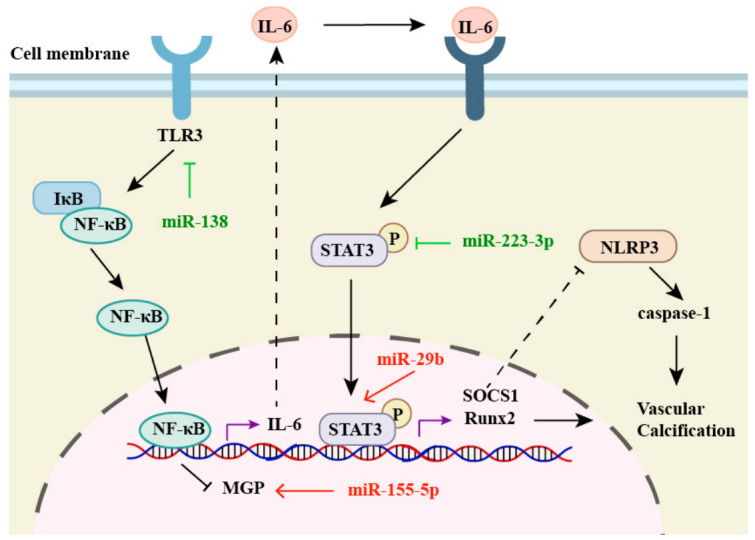
miRNAs targeting the NF-κB inflammatory signaling pathway. In the NF-κB/inflammation signaling pathway, molecular events start from membrane receptor activation and propagate stepwise downstream, with multiple nodes playing promoting or inhibitory roles in vascular calcification. The most upstream part of the pathway includes activation of Toll-like receptor 3 (TLR3), which can initiate downstream NF-κB signaling. Upon activation, NF-κB translocates into the nucleus and induces the transcription of various pro-inflammatory factors, among which key downstream molecules include interleukin-6 (IL-6) and its signal transducer and activator of transcription 3 (STAT3). Excessive activation of the IL-6/STAT3 axis promotes the osteoblast-like phenotypic transformation of VSMCs. Downstream of STAT3, suppressor of cytokine signaling 1 (SOCS1) normally acts as a negative feedback regulator that inhibits NLRP3 inflammasome activation; when SOCS1 expression is downregulated, the NLRP3 inflammasome is activated, inducing pyroptosis and promoting osteogenic differentiation and calcification of VICs. Red miRNAs indicate promotion of vascular calcification; green miRNAs indicate inhibition of vascular calcification. Red arrows indicate activation of the signaling pathway, while green arrows indicate suppression.Black arrows indicate the direction of the signaling pathway.

In the NF-κB inflammatory signaling pathway, several miRNAs regulate vascular or valvular calcification by targeting different nodes of this pathway (Figure 3, Table S1). At the upstream level of the pathway, miR-138 directly binds and inhibits the mRNA of TLR3 (Toll-like receptor 3), blocking the activation of downstream NF-κB signaling, thereby inhibiting vascular calcification [47]. In addition, miR-155-5p is upregulated via the ROS/NF-κB pathway upon induction by the uremic toxin indoxyl sulfate (IS) and promotes vascular calcification by targeting and inhibiting MGP [48]. At the level of downstream inflammatory signals of NF-κB, miR-223-3p blocks the IL-6/STAT3 signaling pathway by targeting and inhibiting STAT3 (a key transcription factor for the downstream pro-inflammatory cytokine IL-6 of NF-κB), thereby preventing VSMCs from transitioning to an osteoblast-like phenotype, exerting a dual protective effect against medial calcification and atherosclerotic calcification. Loss of miR-223-3p significantly exacerbates both types of vascular calcification [49]. In contrast, miR-29b is upregulated in calcific aortic valve disease (CAVD) and, by targeting and inhibiting STAT3, downregulates SOCS1 expression, thereby activating NLRP3 inflammasome-mediated pyroptosis, promoting osteogenic differentiation and calcification of VICs [50]. At the negative feedback regulation level of the NF-κB pathway, miR-34a exacerbates cellular senescence and oxidative stress by targeting and inhibiting SIRT6, thereby weakening the Keap1/NRF2-mediated antioxidant defense (including HO-1, NQO1, GCLC, etc.), thus promoting physiological aging-induced arterial medial calcification; liraglutide reverses this calcification process by downregulating miR-34a expression and restoring the SIRT6 and downstream antioxidant pathways [51].

### 2.4. miRNAs Targeting the MAPK and PI3K/AKT Signaling Pathways

**Figure 4:**
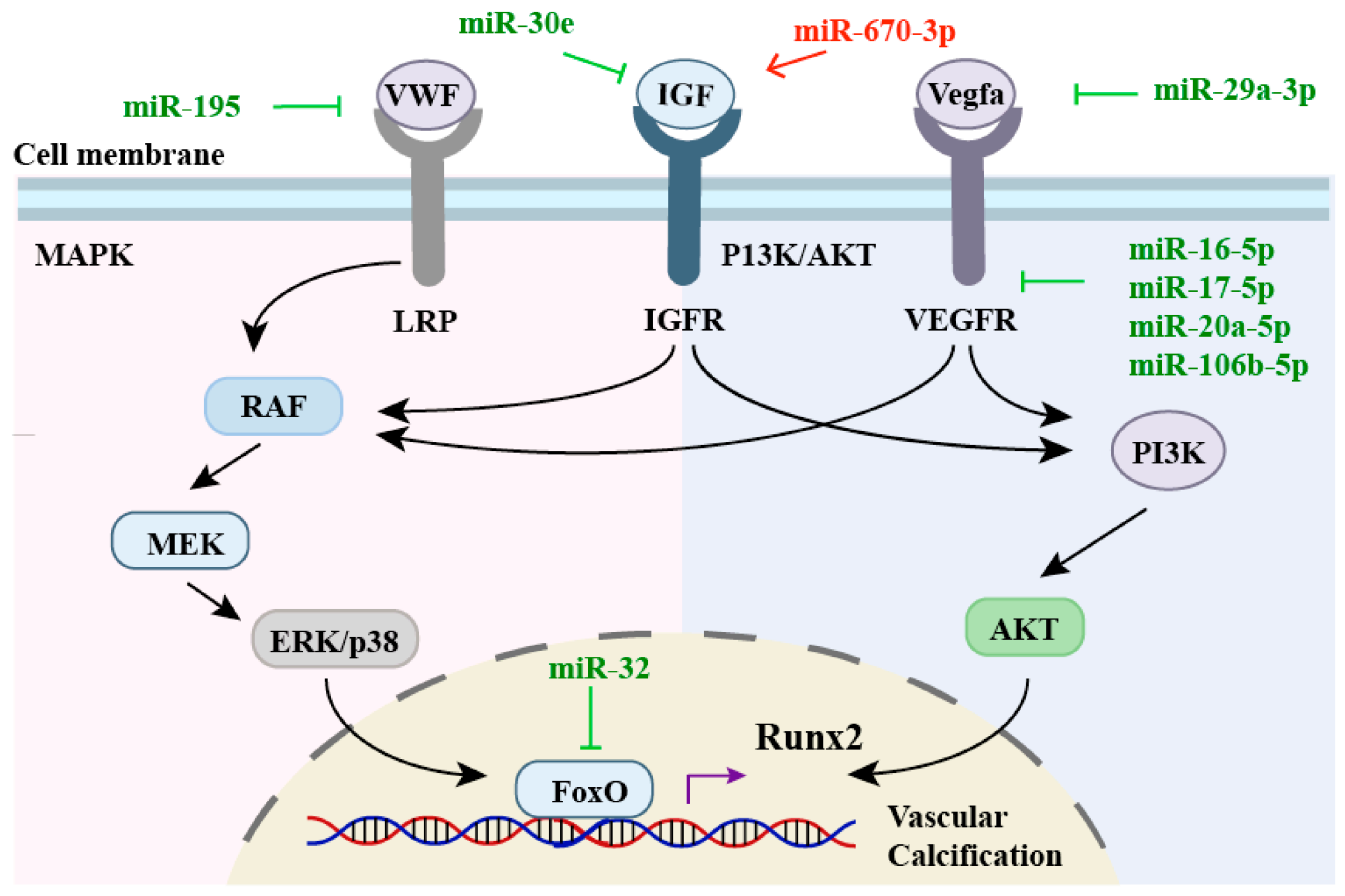
miRNAs targeting the MAPK and PI3K/AKT signaling pathways. In the MAPK signaling pathway, upstream ligands such as VWF, IGF, and VEGF bind to their corresponding receptors (LRP, IGFR) on the cell membrane, sequentially activating intracellular RAF kinase and MEK kinase, ultimately leading to phosphorylation-dependent activation of ERK/p38. Activated ERK/p38 translocate into the nucleus and upregulate the expression of the osteogenic transcription factor Runx2, thereby promoting osteogenic differentiation and calcification of VSMCs or VICs. The PI3K/AKT signaling pathway is also initiated by binding of IGF and VEGF to IGFR, activating PI3K and subsequently phosphorylating and activating AKT. Activated AKT indirectly regulates Runx2 expression levels. Red miRNAs indicate promotion of vascular calcification; green miRNAs indicate inhibition of vascular calcification. Red arrows indicate activation of the signaling pathway, while green arrows indicate suppression. Black arrows indicate the direction of the signaling pathway.

In the MAPK and PI3K/AKT signaling pathways, several miRNAs regulate vascular or valvular calcification by targeting different nodes (Figure 4, Table S1). These two pathways are core signaling axes that respond to growth factors (e.g., IGF, VEGF) and mitogenic stimuli, and they coordinately regulate VSMC proliferation and osteogenic differentiation during vascular calcification. At the upstream growth factor and receptor level, miR-195 is downregulated in CAVD; upregulation of miR-195 alleviates osteogenic differentiation and calcium deposition of VICs by targeting and inhibiting VWF and blocking the p38-MAPK signaling pathway [52]. In addition, miR-30e is highly expressed in normal vessels and inhibits osteogenic differentiation and calcification by suppressing IGF2, while promoting differentiation toward adipogenic or smooth muscle lineages; under aging and atherosclerotic conditions, miR-30e expression is downregulated, leading to increased IGF2 expression and thereby promoting vascular calcification, suggesting that miR-30e is a potential anti-calcification regulator [53]. In a high-phosphate environment, miR-29a-3p significantly alleviates VSMC calcification by targeting and inhibiting Vegfa expression, providing a potential therapeutic target for CKD-related vascular calcification [54]. Meanwhile, circulating small extracellular vesicles from CKD patients exhibit a coordinated deficiency of miR-16-5p, miR-17-5p, miR-20a-5p, and miR-106b-5p, collectively relieving their targeted inhibition of the VEGFA-VEGFR2 signaling axis, thereby driving VSMC osteogenic transformation and vascular calcification; each of these miRNAs has good predictive value for calcification [55]. In contrast, miR-670-3p is enriched in high-phosphate-induced endothelial cell-derived exosomes, and after being taken up by VSMCs, it promotes their osteogenic-like differentiation and calcification by targeting and inhibiting IGF-1; plasma exosomal miR-670-3p levels in end-stage renal disease patients are positively correlated with the degree of coronary artery calcification [56]. Moreover, miR-32 is elevated in the plasma of patients with type 2 diabetes-associated vascular calcification and promotes endothelial cell ferroptosis. Extracellular vesicles derived from bone marrow mesenchymal stem cells alleviate vascular calcification by inhibiting miR-32-mediated ferroptosis and regulating the FoxO/MAPK signaling pathway [57]. Finally, at the level of the PI3K/AKT signaling branch and its cross-regulation with AMPK-mTOR, miR-22 is significantly upregulated in calcified human aortic valves; overexpression of miR-22 directly targets and inhibits CAB39 expression, thereby weakening the catalytic activity of the CAB39-LKB1-STRAD complex, inhibiting the AMPK-mTOR signaling pathway, and promoting osteogenic differentiation and calcification of VICs [58].

### 2.5. miRNAs Targeting the Endoplasmic Reticulum (ER) Stress and Autophagy Pathways

In the ER stress and autophagy pathways, several miRNAs regulate vascular or valvular calcification by targeting different nodes of these pathways, mostly exerting inhibitory effects. The unfolded protein response of ER stress is mediated by three parallel sensors: PERK, IRE1, and ATF6. At the ATF6 branch, miR-199a-5p is significantly downregulated in CAVD; overexpression of miR-199a-5p directly targets and inhibits ATF6 and GRP78, thereby blocking activation of this branch and suppressing osteogenic differentiation and calcification of VICs [59]. At the PERK/eIF2α/ATF4 branch, miR-4731-5p is downregulated in β-glycerophosphate-induced VSMC calcification; overexpression of miR-4731-5p inhibits PERK/ATF4-mediated ER stress, thereby alleviating osteogenic-like phenotypic transformation and calcium deposition [60]. miR-129 and miR-342, delivered via exosomes from intermittent hypoxia-stimulated VSMCs to VICs, cooperatively target and inhibit the eIF2α/ATF4 signaling axis, thereby attenuating osteogenic-like differentiation of VICs and progression of CAVD [61]. At the downstream level of the pathway, miR-214 is significantly downregulated in calcified aortic valve tissues; overexpression of miR-214 directly targets and inhibits ATF4 and Sp7, suppressing osteogenic differentiation and calcification of VICs, while miR-214 knockout exacerbates aortic valve calcification [62]. As a downstream effector mechanism of ER stress, the autophagy pathway is also involved in calcification regulation. Cold exposure-induced plasma exosomes are enriched in miR-320a-3p, which activates the AMPK/mTOR autophagy pathway by targeting and inhibiting PDCD4, thereby suppressing osteogenic differentiation and calcification of VSMCs [63]. Conversely, miR-181c-5p is upregulated in CKD-associated vascular calcification and, by targeting and inhibiting SIRT1, inhibits Pink1/Parkin-mediated mitophagy and promotes ferroptosis, thereby driving osteogenic-like differentiation and calcification of VSMCs [64].

### 2.6. miRNAs Targeting the Notch Signaling Pathway

In the Notch signaling pathway, miR-29a-5p and miR-34b target upstream and downstream nodes of the pathway, respectively, to exert regulatory effects. In the context of CKD, parathyroid hormone downregulates miR-29a-5p expression and, by relieving the inhibition of its target gene GSAP, activates the γ-secretase/Notch1 signaling pathway, thereby inducing valvular endothelial-to-mesenchymal transition and promoting valve calcification [65]. In contrast, miR-34b inhibits VSMC differentiation toward osteoblast-like cells and thus suppresses vascular calcification by targeting its target gene Notch1. Under high-phosphate conditions, miR-34b expression is suppressed at the cellular level and animal level and in uremic patients and is negatively correlated with the degree of calcification [66].

### 2.7. miRNAs Targeting the Extracellular Matrix and Calcification Inhibitors

In the regulatory network of the extracellular matrix and calcification inhibitors, several miRNAs exert inhibitory or promoting effects on vascular and valvular calcification by targeting different nodes. On the inhibitory side, miR-29b-3p directly targets and inhibits matrix metalloproteinase 2 (MMP2) expression, thereby suppressing the vascular calcification process [67]. On the promoting side, miR-874-3p promotes vascular calcification by directly targeting and inhibiting the anti-calcification gene Adam19. Adam19 exerts an anti-calcification effect mainly by maintaining the normal contractile phenotype of VSMCs and preventing their transformation into osteoblast-like cells [68]. In addition, miR-143-3p promotes vascular calcification by targeting and inhibiting matrix Gla protein (MGP); under high-glucose conditions, it is sponged by MALAT1 in macrophage-derived exosomes, thereby relieving the inhibition of MGP and attenuating VSMC calcification [69]. Furthermore, miR-181b is upregulated in valvular fibrosa layer endothelial cells under oscillatory shear stress; it enhances matrix metalloproteinase activity by targeting and inhibiting TIMP3, promoting extracellular matrix degradation, and thus participates in the early pathological remodeling and progression of CAVD [70].

### 2.8. Circulating miRNAs as Potential Biomarkers for Vascular and Valvular Calcification

Accumulating evidence indicates that altered expression of circulating miRNAs in various disease contexts makes them potential non-invasive biomarkers for vascular and valvular calcification (Table 1). In vascular calcification associated with CKD, end-stage renal disease (ESRD), and dialysis, several circulating miRNAs are downregulated and may serve as potential biomarkers, including miR-106a-5p [71], miR-223-3p and miR-93-5p [20], miR-211-5p [21], miR-31-5p [72], miR-125b [19], miR-143-5p [73], and miR-145 and miR-486 [74]. Among these, miR-106a-5p is negatively correlated with calcification severity and serum PTH and osteocalcin levels; miR-223-3p and miR-93-5p are downregulated in CKD stage 4-5 and recover after renal transplantation; miR-211-5p gradually decreases with calcification progression; low miR-31-5p expression is an independent risk factor; miR-125b synergizes with osteoprotegerin to predict risk; circulating miR-143-5p levels are reduced, and its overexpression inhibits calcification; miR-145 and miR-486 are downregulated in both calcified arteries and serum, and serum miR-145 is an effective biomarker. In patients with arterial calcification, circulating miR-126-3p and miR-145-5p are significantly downregulated, with low miR-126-3p expression predicting major adverse cardiovascular events (MACE) [75]. In CAVD, miR-17-5p and miR-29a-3p [76] are downregulated in serum and are consistent with changes in valve tissue; low baseline plasma miR-223 [77] expression is associated with increased risk of adverse events after transcatheter aortic valve implantation; miR-210 [78] upregulation is independently associated with increased mortality, while miR-92a is highly expressed in bicuspid aortic valves and positively correlated with transvalvular pressure gradient [79].

However, it must be acknowledged that current circulating miRNA biomarker studies in vascular calcification face several critical limitations, including small or unreported cohort sizes, lack of independent validation cohorts, absence of standardized normalization protocols, insufficient reporting of ROC/AUC performance, sensitivity, and specificity, inadequate control for clinical confounders such as CKD staging, dialysis status, and concomitant medications, and potential hemolysis effects that were rarely systematically assessed. These issues must be addressed in future multicenter studies with standardized protocols and comprehensive confounder adjustment before clinical translation can be realized.

### 2.9. Context-Dependent miRNAs (Functional Duality)

The functional duality of miRNAs arises from their intrinsic ability to regulate multiple target genes via seed-sequence complementarity, combined with context-dependent variations in target gene expression, signaling pathway activity, and cellular microenvironment. This means that the same miRNA can exert opposite regulatory effects in different disease contexts by targeting different genes, as exemplified by the following cases.

The same miRNA can exert opposite regulatory effects in different disease contexts by targeting different genes, as exemplified by miR-21-5p (Table 2). In CAVD, miR-21-5p promotes osteogenic differentiation and calcification of VICs by targeting and inhibiting transforming growth factor-β-induced protein (TGFBI) [12]. In CKD-associated vascular calcification, exosomes secreted by adventitial fibroblasts are enriched in miR-21-5p; after uptake by VSMCs, they target and inhibit Crim1, thereby promoting osteogenic-like differentiation and calcification [13]. In diabetic vascular calcification, miR-21-5p promotes VSMC osteogenic differentiation and calcification by targeting and inhibiting Tpm1; the Danlian–Tongmai formula combined with intermittent fasting exerts anti-calcification effects by inhibiting the miR-21-5p/Tpm1 axis [14]. These studies indicate that the pro-calcific function of miR-21-5p is context-dependent, with the specific mechanism depending on the targeted gene and the disease microenvironment.

The same miRNA may exhibit completely different expression changes and functional effects in different disease contexts, as exemplified by miR-155-5p (Table 2). Under normal conditions, miR-155-5p exerts an anti-vascular calcification effect by targeting and inhibiting RCN2. However, under high-phosphate conditions, its expression is significantly downregulated due to negative regulation by the RCN2/STAT3 axis, forming a vicious RCN2/STAT3/miR-155-5p feedback loop that weakens the protective effect of miR-155-5p, thereby driving VSMC osteogenic-like differentiation and calcification [80]. Under induction by the uremic toxin IS, miR-155-5p is upregulated via the ROS/NF-κB pathway and promotes vascular calcification by targeting and inhibiting MGP [48]. Furthermore, in human peripheral arterial calcification lesions, miR-155 expression is upregulated, and its overexpression promotes VSMC osteogenic differentiation and mineralization; this pro-calcific effect may be mediated by targeting and inhibiting CD73 and Smad3, and among three miRNAs (miR-136, miR-155, miR-183), miR-155 shows the most significant effect [81]. These results indicate that miR-155-5p function is strongly context-dependent, with its direction of action being co-regulated by the disease microenvironment, upstream signaling pathways, and the target gene repertoire.

The same miRNA can exert opposite effects in different disease contexts, as exemplified by miR-145-5p (Table 2). In vascular calcification, miR-145-5p directly binds and inhibits the expression of the pro-calcific key gene Smad3, preventing VSMC differentiation toward osteoblast-like cells, thereby inhibiting vascular calcification [42]. In contrast, in CAVD, miR-145-5p is enriched in extracellular vesicles and promotes osteogenic differentiation and calcification of VICs by targeting and inhibiting ZEB2, thereby upregulating ALPL expression [82]. These results indicate that miR-145-5p function is tissue- or disease-context-dependent, with its direction of action depending on the targeted gene and cellular microenvironment.

## 3. Regulatory Mechanisms and Functional Heterogeneity of lncRNAs

### 3.1. lncRNAs That Inhibit Vascular Calcification

#### 3.1.1. ceRNA (miRNA Sponging) Class of Anti-Calcific lncRNAs

Among lncRNAs that inhibit vascular calcification, the ceRNA mechanism is the most common class (Table 3). ceRNA (competing endogenous RNA) acts by sponging miRNAs, preventing them from binding to their target mRNAs, thereby upregulating the expression of protective target genes. In high-phosphate-induced VSMC calcification, lncRNA HOTAIR attenuates calcification by targeting and inhibiting miR-126 to upregulate Klotho expression, thereby activating SIRT1 and blocking the Wnt/β-catenin signaling pathway [83]. Similarly, in a high-phosphate-induced model, lncRNA GAS5 expression is downregulated; overexpression of GAS5 upregulates PTEN expression by sponging miR-26b-5p, thereby inhibiting osteogenic differentiation and calcification [84]. In addition, lncRNA SNHG29 is also downregulated in high-phosphate-induced VSMC calcification; its overexpression activates the α-Klotho/FGFR1/FGF23 axis by sponging miR-200b-3p, inhibiting osteogenic differentiation and calcification [85]. In valve calcification, lncRNA OIP5-AS1 is downregulated in osteogenically induced VICs and upregulates TWIST1 expression by acting as a molecular sponge for miR-137, exerting a protective effect against VIC osteogenic differentiation [86]. lncRNA FGD5-AS1 is downregulated in CAVD and upregulates BIRC5 expression by sponging miR-497-5p, thereby inhibiting VIC osteogenic differentiation and alleviating aortic valve calcification [87].

#### 3.1.2. Direct Protein-Binding and Epigenetic Regulation Class of lncRNAs

Among lncRNAs that inhibit vascular calcification, some molecules act through direct protein binding or epigenetic regulation rather than the ceRNA mechanism (Table 3). lncRNA SNHG1 alleviates high glucose-induced VSMC calcification and senescence by stabilizing Bhlhe40 mRNA and promoting its SUMOylation and nuclear translocation, thereby inhibiting the expression of the autophagy key gene Atg10 [88]. lncRNA HOTAIR, transcriptionally activated by the transcription factor E2F1, acts as a molecular scaffold to recruit KDM1A and EZH2 to epigenetically silence KLF16, thereby relieving the transcriptional inhibition of Klotho by KLF16 and inhibiting high-phosphate-induced vascular calcification [89].

#### 3.1.3. lncRNAs That Inhibit Osteogenic Signaling Pathways

Some lncRNAs exert anti-calcification effects by directly inhibiting osteogenic gene expression or blocking osteogenic signaling pathways (Table 3). lncRNA Lrrc75a-as1 is downregulated in high-phosphate-induced VSMC calcification; overexpression of Lrrc75a-as1 alleviates calcium deposition by inhibiting the expression of osteogenic markers such as Runx2, Msx2, and BMP2, while knockdown exacerbates calcification, suggesting a protective role as a negative regulator of vascular calcification [90]. lncRNA-EPS alleviates high glucose-induced VSMC osteogenic-like differentiation and calcium deposition by inhibiting the inflammatory response and blocking the Wnt/β-catenin signaling pathway, thereby ameliorating diabetes-associated vascular calcification [91].

### 3.2. lncRNAs That Promote Vascular Calcification

#### 3.2.1. ceRNA (miRNA Sponging) Class of Pro-Calcific lncRNAs

Among lncRNAs that promote vascular calcification, the ceRNA mechanism is also common, acting by sponging miRNAs to upregulate the expression of osteogenic target genes (Table 3). In valve calcification, lncRNA NEAT1 upregulates PTEN expression by acting as a molecular sponge for miR-214-3p, thereby inhibiting the PI3K/AKT/mTOR signaling pathway and promoting VIC osteogenic differentiation, thus driving CAVD progression [92]. lncRNA TUG1 is upregulated in calcified human aortic valves and VICs and upregulates Runx2 expression by sponging miR-204-5p, thereby promoting VIC osteogenic differentiation and calcification [93]. During vascular calcification, lncRNA MALAT1 expression increases and promotes VSMC calcification by sponging miR-30c to upregulate its target gene Runx2 [15]. In a high-phosphate-induced VSMC calcification model, lncRNA Prrc2c promotes vascular calcification by sponging miR-145-5p to upregulate Smad3 expression [42]. Similarly, in a high-phosphate-induced model, lncRNA SENCR promotes VSMC osteogenic differentiation and calcification by sponging miR-4731-5p, thereby relieving the latter’s inhibition of ATF4, activating the PERK/ATF4-mediated ER stress pathway, and upregulating Runx2 expression [60]. In addition, in adipose-derived mesenchymal stem cells, MALAT1 also acts as a ceRNA, sponging miR-30 to relieve its inhibition of Runx2, thereby promoting the expression of osteogenic genes (such as OCN, OPN, OSX) and osteogenic differentiation [33].

#### 3.2.2. Epigenetic and Transcriptional Regulation Class of lncRNAs

Among lncRNAs that promote vascular calcification, some molecules directly bind the epigenetic regulator EZH2 to regulate gene transcription, thereby accelerating the calcification process (Table 3). In vascular calcification, lncRNA NEAT1 directly binds EZH2, promoting its translocation from the cytoplasm to the nucleus, thereby inhibiting the transcription of senescence- and migration-related genes (such as P16, P21, TIMP3), thus enhancing VSMC proliferation, migration, and osteogenic differentiation, accelerating the pathological progression of vascular calcification [16]. In valve calcification, lncRNA SNHG3 is upregulated in calcified human aortic valves and, by interacting with EZH2, inhibits H3K27 trimethylation in the BMP2 promoter region, thereby upregulating BMP2 expression and activating its signaling pathway, promoting VIC osteogenic differentiation and calcification [94].

#### 3.2.3. Signaling Pathway, Inflammation, and Phenotypic Transformation Class of lncRNAs

Among lncRNAs that promote vascular calcification, some exert pro-calcific effects by regulating classical signaling pathways, the inflammatory microenvironment, intercellular communication, or phenotypic transformation (Table 3). In terms of signaling pathway and autophagy regulation, lncRNA MEG3 induces VIC osteogenic differentiation by activating the Wnt/β-catenin signaling pathway, thereby promoting the occurrence and progression of aortic valve calcification [95]. lnc-COL6A1-6 is significantly upregulated in calcified aortic valve tissues and during VIC osteogenic differentiation; knockdown of lnc-COL6A1-6 alleviates VIC osteogenic differentiation and calcium deposition by inhibiting autophagy activity [96]. In terms of inflammation and oxidative stress regulation, lncRNA AFAP1-AS1 is upregulated in calcified human aortic valves and promotes M1 macrophage polarization while inhibiting M2 polarization, thereby exacerbating VIC osteogenic differentiation and promoting the progression of aortic valve calcification [97]. lncRNA FAS-AS1 is highly expressed in the serum of ESRD and vascular calcification patients and has diagnostic value; silencing FAS-AS1 inhibits high-phosphate-induced VSMC calcification by reducing reactive oxygen species levels and suppressing the expression of tumor necrosis factor-α and interleukin-6, thereby alleviating oxidative stress and inflammation [98]. In terms of intercellular communication and phenotypic transformation, lincRNA-p21 is upregulated in high-phosphate-induced VSMC calcification and, via small extracellular vesicle-mediated pro-calcific signaling, promotes VSMC osteogenic transdifferentiation, apoptosis, and calcium deposition, while knockdown of lincRNA-p21 significantly alleviates these calcification phenotypes [99]. lncRNA ANRIL (encoded by the 9p21.3 chromosomal locus—the gene desert region most strongly associated with coronary artery disease to date) drives the transition of VSMCs to an osteochondrogenic-like state in a cell-autonomous manner in VSMCs carrying risk haplotypes, thereby promoting arterial calcification [100].

#### 3.2.4. Context-Dependent lncRNAs

lncRNA H19 drives vascular calcification by activating different downstream pathways in different pathophysiological contexts (such as CKD, diabetes, and high-phosphate environment), exhibiting functional context dependence (Table 3). In a CKD-associated high-phosphate environment, H19 acts as a “molecular sponge” for microRNA-138, relieving its inhibition of the TLR3 gene, thereby activating the NF-κB signaling pathway and ultimately exacerbating vascular calcification [47]. In a diabetic model, H19 exacerbates vascular calcification by inhibiting the BI-1/OPA1 protein pathway, enhancing calcium deposition, osteogenic-like differentiation, and apoptosis of VSMCs [101]. In addition, in high-phosphate-induced VSMC calcification, H19 expression is upregulated due to hypomethylation of its promoter CpG island, thereby promoting osteogenic-like differentiation and calcium deposition by activating the Erk1/2 signaling pathway [102].

## 4. Unique Regulatory Modes of circRNAs

circRNAs exert inhibitory effects in vascular calcification, valvular calcification, and vascular injury-related pathologies through multiple mechanisms (Table 4). In vascular calcification, circSmoc1-2 inhibits VSMC calcification by sponging miR-874-3p to indirectly upregulate its target gene Adam19 [68]. In CAVD, circZBTB44 expression is downregulated; it translates a novel peptide ZBTB44-342aa via m^6^A modification, which binds IGF2BP3 to stabilize CAMK1, thereby inhibiting mitochondrial damage and mtDNA leakage, subsequently blocking cGAS-STING pathway activation, and alleviating VIC osteogenic differentiation and calcification [18]. Also in valve calcification, circHIPK3, via DDX5/METTL3-mediated m^6^A modification, recruits IGF2BP2 to stabilize Krm1 mRNA, enhancing the inhibitory effect of the Krm1-Dkk2 complex on the Wnt/β-catenin pathway, thereby antagonizing VIC osteogenic differentiation and aortic valve calcification; miR-182-5p synergistically weakens this protective effect by targeting and inhibiting Dkk2 [23]. In the context of vascular injury and atherosclerosis, circSMAD3 expression is downregulated; it acts as a molecular scaffold to enhance the interaction between hnRNPA1 and the E3 ubiquitin ligase WDR76, promoting ubiquitination and degradation of hnRNPA1, thereby regulating p53 pre-mRNA splicing and activating the p53γ signaling pathway, inhibiting VSMC proliferation and phenotypic switching, and exerting a potential protective effect against vascular calcification [103].

In terms of promoting vascular and valvular calcification, circRNAs also act through multiple mechanisms (Table 4). In vascular calcification, circ-GALK2 is significantly upregulated in β-glycerophosphate-induced calcified VSMCs and promotes VSMC osteogenic-like differentiation and calcification by acting as a molecular sponge for miR-134-3p to upregulate CD36 expression; plasma circ-GALK2 levels are positively correlated with the degree of aortic calcification [17]. circTOP1 promotes VSMC transition to an osteoblast-like phenotype by targeting and regulating PTBP1 expression, thereby exacerbating the progression of coronary artery calcification [104]. In addition, circ_0008362 is enriched in high-glucose-induced endothelial cell-derived extracellular vesicles; after being taken up by VSMCs, it both sponges miR-1251-5p to upregulate Runx2 expression and directly interacts with Runx2 protein, synergistically promoting VSMC osteogenic differentiation and calcification; plasma EV circ_0008362 levels are positively correlated with the severity of coronary and aortic calcification in diabetic patients [105]. In valve calcification, circARHGAP10 is highly expressed and sponges miR-335-3p, relieving its inhibition of RUNX2, thereby upregulating RUNX2 expression and driving VIC osteogenic-like differentiation and calcium deposition [106]. circ-CCND1 is highly expressed in CAVD and upregulates CCND1 by sponging miR-138-5p, thereby activating the CCND1/P53/P21 signaling pathway, promoting VIC osteogenic differentiation and calcium deposition [107]. CircRIC3 promotes VIC osteogenic differentiation and aortic valve calcification by acting as a molecular sponge for miR-204-5p to upregulate DPP4 expression; melatonin alleviates calcification by downregulating the CircRIC3/miR-204-5p/DPP4 signaling axis [108].

## 5. Other Novel Non-Coding RNAs Associated with Calcification

In addition to miRNAs, lncRNAs, and circRNAs, novel non-coding RNAs such as PIWI-interacting RNAs (piRNAs) and tRNA-derived small RNAs (tsRNAs) are also involved in the regulation of vascular and valvular calcification. In aortic valve calcification, AVCAPIR (aortic valve calcification-associated PIWI-interacting RNA) is significantly upregulated and directly binds to and inhibits the m^6^A demethylase activity of FTO, thereby enhancing the m^6^A modification and IGF2BP1-dependent stability of CD36 mRNA, subsequently upregulating the CD36/PCSK9 signaling axis and promoting VIC osteogenic differentiation and aortic valve calcification [109]. In addition, tsRNA-5006c is enriched in M1 macrophage-derived extracellular vesicles; after being taken up by VICs in CAVD, it promotes their osteogenic differentiation and calcification by enhancing mitophagy [110].

## 6. Discussion

### 6.1. Microenvironment-Dependent Regulatory Mechanisms of Non-Coding RNA Functional Duality

The functional duality of non-coding RNAs is a phenomenon worthy of in-depth discussion in the regulatory network of vascular calcification, with its roots in differences in the cellular microenvironment and target gene repertoire. Taking miR-21-5p as an example, it promotes VIC osteogenic differentiation by targeting TGFBI in CAVD [12], drives VSMC calcification via exosome delivery and targeting of Crim1 in CKD [13], and exerts pro-calcific effects by inhibiting Tpm1 in the diabetic context [14]. Similarly, miR-155-5p is upregulated via the ROS/NF-κB pathway under uremic toxin induction and promotes calcification by inhibiting MGP [48], but it has also been shown to have a significant pro-calcific effect in a study of human peripheral arterial calcification [81]. miR-145-5p inhibits Smad3 to exert anti-calcification effects in vessels [42] but promotes calcification in valves by inhibiting ZEB2 to upregulate ALPL [82]. lncRNA H19 also activates distinctly different downstream pathways such as NF-κB and Erk1/2 or inhibits BI-1/OPA1 in different environments (high phosphate, diabetes, etc.) [47,101,102]. These functional heterogeneities suggest that the same non-coding RNA can exert opposite effects through different “RNA-target gene-signaling pathway” modules depending on cell type (VSMC vs. VIC), disease state (uremia, diabetes, aging), and extracellular signals (phosphate, inflammatory cytokines, mechanical forces). This also poses challenges for the development of RNA therapeutics—when designing oligonucleotide drugs (such as antagomirs, siRNAs, or ASOs), disease-specific targeted delivery must be carefully considered to avoid off-target effects and functional reversal risks from systemic administration. Systemic delivery of miRNA inhibitors or mimics could inadvertently affect healthy tissues where the same miRNA serves essential physiological functions, potentially causing unintended toxicities such as immune activation, hepatotoxicity, or disruption of normal tissue homeostasis. In recent years, technologies such as GalNAc-modified siRNAs and engineered exosome-targeted delivery have provided new possibilities for achieving tissue- or cell-selective non-coding RNA interventions, which is a key technical direction to address the functional duality issue and minimize off-target toxicity in the future.

However, the field currently lacks consensus regarding the conflicting conclusions observed for the same ncRNA across different studies—for example, the same miRNA has been reported to promote calcification in some models while inhibiting it in others. These discrepancies may arise from multiple factors, including differences in cell types (primary VSMCs vs. immortalized cell lines), disease models (in vitro high-phosphate stimulation vs. CKD animal models vs. diabetic models), detection methods (calcification quantification approaches, ncRNA overexpression/knockdown strategies), and experimental conditions (stimulus concentrations, time windows), all of which can influence the final functional output of a given ncRNA. Importantly, most current studies have examined ncRNA functions in a single experimental context, lacking longitudinal investigations that systematically validate the role of the same ncRNA across multiple models. Thus, some conflicting findings may reflect genuine biological differences, while others may be partially attributable to variations in experimental systems or inter-study heterogeneity. Future studies that systematically evaluate the same ncRNA across multiple cell types, disease models, and stimulus conditions will be essential to distinguish its true physiological relevance from context-dependent artifacts.

### 6.2. Targeted Intervention Strategies Against Pro-Calcific Non-Coding RNAs and Insights for Drug Translation

The strategies summarized in this review indicate that targeted inhibition of pro-calcific non-coding RNAs or rescue of their downstream pathways are effective approaches to ameliorate vascular calcification. Melatonin alleviates VIC osteogenic differentiation by downregulating the CircRIC3/miR-204-5p/DPP4 signaling axis [108]; liraglutide reverses aging-associated medial calcification by downregulating miR-34a to restore the SIRT6/NRF2 antioxidant pathway [51]; and the Danlian–Tongmai formula combined with intermittent fasting exerts anti-diabetic vascular calcification effects by inhibiting the miR-21-5p/Tpm1 axis [14]. These findings reveal the great potential of non-coding RNAs as “druggable targets.” Currently, small-molecule drugs, natural products, and even metabolic regulation approaches can indirectly intervene in diseases by affecting the expression or function of non-coding RNAs. Particularly in the context of rapid advances in RNA therapeutics, antisense oligonucleotides (ASOs) and small interfering RNAs (siRNAs) targeting pro-calcific non-coding RNAs will gradually enter the cardiovascular field. For example, designing GapmeR ASOs targeting pro-calcific lncRNAs (such as NEAT1 or MALAT1) or circRNAs or using lipid nanoparticles (LNPs) or extracellular vesicles to encapsulate anti-calcific miRNAs holds promise for precision inhibition. In the future, combining small-molecule drugs with RNA-targeted therapies may bring new paradigms for the precision treatment of vascular calcification.

### 6.3. Clinical Translation Prospects of Protective Non-Coding RNA Replacement Therapy

Non-coding RNAs as therapeutic agents themselves have achieved breakthroughs in several fields, and protective miRNAs in cardiovascular calcification also have clinical translation potential. miR-133a, a classic anti-calcific miRNA, alleviates VSMC calcification by inhibiting Runx2 and osteocalcin [10], and circulating miR-133a levels can predict the reversibility of left ventricular hypertrophy after aortic valve replacement [111]. More inspiringly, a recent study used genetically engineered extracellular vesicles displaying hydroxyapatite-binding peptides on their surface to achieve targeted delivery of miR-133a to calcified vessels, effectively reducing vascular calcification in an atherosclerotic mouse model [112]. This fully demonstrates the feasibility of RNA replacement therapy in cardiovascular calcification. With the approval of the siRNA drug inclisiran for lipid-lowering therapy as a representative example, RNA therapeutics have achieved clinical translation breakthroughs in the cardiovascular field [113], providing an important reference for developing protective miRNA mimics for the treatment of vascular calcification. Developing delivery systems for protective miRNA mimics (such as miR-133a, miR-204, miR-30e) based on lipid nanoparticles or exosomes has become a highly promising translational direction. Future challenges lie in further improving targeting efficiency, prolonging half-life, reducing immunogenicity, and selecting optimal delivery strategies according to the type of calcification (medial calcification, valvular calcification).

### 6.4. Non-Coding RNA Co-Regulatory Networks and Multi-Target Combination Intervention Strategies

Non-coding RNAs do not function in isolation; their co-regulatory networks provide a theoretical basis for multi-target combination intervention. Studies have found a coordinated deficiency of miR-17-5p, miR-20a-5p, and miR-106b-5p in circulating small extracellular vesicles from CKD patients, collectively relieving inhibition of the VEGFA-VEGFR2 axis and driving VSMC osteogenic transformation [55]; in a Klotho-deficient environment, miR-135a, miR-762, miR-714, and miR-712 are coordinately upregulated, leading to intracellular calcium overload and calcification by simultaneously inhibiting multiple calcium efflux proteins [114]. These co-regulatory patterns suggest that single RNA intervention may have limited efficacy due to compensatory mechanisms. In the future design of RNA therapeutics, multi-target combination strategies—such as simultaneously using mimics of several protective miRNAs, combining siRNAs/ASOs targeting different pro-calcific pathways, or developing circular RNA “super-sponges” that can simultaneously regulate multiple miRNAs—may achieve more comprehensive and durable anti-calcification effects. In addition, the combination of non-coding RNAs with conventional drugs (such as phosphate binders, SGLT2 inhibitors, etc.) may also exert synergistic protective effects and deserves further validation in preclinical studies.

### 6.5. Safety Considerations and Off-Target Effects in ncRNA Therapeutics

The therapeutic application of non-coding RNAs, whether as inhibitors (antagomirs, ASOs, siRNAs) or replacements (miRNA mimics), carries inherent risks of off-target effects that must be carefully evaluated during preclinical and clinical development. Unlike small-molecule drugs with well-characterized pharmacophores, oligonucleotide therapeutics can affect multiple transcripts through partial seed-sequence complementarity, potentially disrupting gene regulatory networks in tissues unrelated to the target pathology. For instance, miR-21 is a ubiquitously expressed microRNA that plays critical roles in the pathogenesis of cancer, fibrosis, cardiovascular diseases, and immune disorders [115]; systemic inhibition of miR-21 to treat vascular calcification could inadvertently promote tumorigenesis or exacerbate cardiac remodeling. To mitigate these risks, several strategies have emerged. Tissue-specific delivery systems, such as lipid nanoparticles conjugated with targeting ligands, can concentrate therapeutic payloads at disease sites while sparing healthy organs [116]. Local administration routes (e.g., perivascular or intracoronary delivery) offer another approach to minimize systemic exposure [117]. Additionally, chemical modifications (e.g., 2′-O-methyl, locked nucleic acids, phosphorothioate backbones) can enhance target specificity and reduce immune stimulation [118,119]. Preclinical safety pharmacology studies should include comprehensive toxicogenomic profiling to identify off-target gene expression changes across multiple organ systems, and clinical trials should incorporate rigorous monitoring for adverse events related to immune activation, hepatotoxicity, and hematological abnormalities.

### 6.6. High-Priority ncRNAs for Therapeutic and Diagnostic Development

Based on evidence strength (human/animal validation), pathological breadth, and mechanistic uniqueness, eight ncRNAs emerge as high-priority candidates. miR-21-5p exemplifies context-dependent duality, promoting calcification via distinct targets across CAVD (TGFBI) [12], CKD (Crim1) [13], and diabetes (Tpm1) [14], with its inhibition showing therapeutic efficacy [14]. miR-133a is a classic anti-calcific miRNA that alleviates VSMC calcification by downregulating Runx2 and osteocalcin, and circulating miR-133a levels can predict the reversibility of left ventricular hypertrophy after aortic valve replacement [10,111]. miR-204-5p exerts consistent anti-calcific effects across VSMCs and VICs by targeting Runx2 [27,30,31,35]. miR-223-3p offers dual protection against medial and atherosclerotic calcification via STAT3 targeting and serves as a circulating biomarker [20,49]. circ_0008362 employs a unique dual mechanism—sponging miR-1251-5p and directly binding Runx2—with plasma levels correlating with calcification severity in diabetic patients [105]. NEAT1 promotes calcification through ceRNA and EZH2-mediated epigenetic mechanisms, making it a prime ASO target [16,92]. H19 displays context-dependent pro-calcific effects across CKD, diabetes, and high-phosphate environments via distinct pathways (NF-κB, BI-1/OPA1, Erk1/2) [47,101,102]. circZBTB44 encodes a peptide that inhibits cGAS-STING signaling, opening avenues for peptide-based therapy [18]. These prioritized ncRNAs, summarized in Table 5, warrant further translational investigation.

### 6.7. Functional Hierarchy of ncRNA Regulation: Genuine Drivers or Downstream Manifestations?

A critical question is whether ncRNA–target axes are genuinely rate-limiting drivers of vascular calcification or downstream manifestations of broader epigenetic and transcriptional reprogramming. Based on available evidence, both scenarios appear to coexist. On one hand, a subset of ncRNAs operate at the upper levels of the regulatory hierarchy: NEAT1 directly binds EZH2 and promotes its nuclear translocation to epigenetically silence P16, P21, and TIMP3, thereby driving VSMC proliferation and osteogenic differentiation [16]; similarly, HOTAIR is transcriptionally activated by E2F1 and acts as a scaffold to recruit KDM1A and EZH2, leading to epigenetic silencing of KLF16 and subsequent depression of Klotho [89]. These examples place certain ncRNAs upstream of epigenetic remodeling, endowing them with genuine “driver” characteristics. On the other hand, many ncRNA–target interactions—particularly those targeting Runx2 and Smad pathways—likely function at intermediate network layers. Runx2 is a well-established osteogenic transcription factor routinely measured as a differentiation marker in virtually all vascular calcification studies; however, it is itself subject to complex regulation at multiple levels—transcriptional control, DNA methylation, histone modifications, and post-translational modifications—making it a downstream readout of multiple upstream signals rather than a solitary switch. Thus, many ncRNAs that converge on Runx2 or Smad may act as signal amplifiers or fine-tuners that modulate the intensity of osteogenic signaling, rather than serving as autonomous initiators of calcification. This distinction has direct therapeutic implications: if most ncRNA–target interactions are downstream of epigenetic reprogramming, single-ncRNA targeting may be insufficient to reverse established calcification unless upstream drivers are also addressed. However, most current studies are limited to linear descriptions of single ncRNA–target axes and lack systematic analyses of functional hierarchies, leaving this question open for future investigation.

## 7. Conclusions

This review establishes a pathway-level framework for understanding ncRNA regulation in vascular calcification, with the following conclusions:

### 7.1. A Pathway-Centric Regulatory Architecture of miRNA Functions

Categorizing miRNAs by their targeted signaling cascades—Wnt/β-catenin, BMP/Smad, NF-κB, MAPK/PI3K/AKT, ER stress/autophagy, and Notch—reveals that miRNAs act as multi-node modulators with extensive cross-talk among pathways. Notably, a subset of ncRNAs (e.g., miR-21-5p, miR-155-5p, miR-145-5p, and lncRNA H19) exhibit context-dependent functional duality, exerting opposing effects depending on cell type, disease setting, or microenvironment. This observation highlights the need for careful target validation and disease-selective delivery in RNA therapeutics.

### 7.2. Mechanistic Heterogeneity Beyond miRNAs

lncRNAs operate through three major modes—ceRNA sponging, epigenetic regulation via EZH2 binding, and direct signaling modulation—often converging on Runx2 and the Wnt/β-catenin pathway. Circular RNAs add further mechanistic layers through peptide encoding (circZBTB44), m^6^A-dependent mRNA stabilization (circHIPK3), molecular scaffolding (circSMAD3), and dual sponging/protein-binding mechanisms (circ_0008362), while emerging piRNAs (AVCAPIR) and tsRNAs (tsRNA-5006c) point to unexplored regulatory territories.

### 7.3. Safety and Combination Strategies as Translational Prerequisites

Systemic ncRNA therapeutics carry inherent risks of off-target effects, immune activation, and hepatotoxicity, underscoring the need for tissue-specific delivery systems (engineered exosomes, LNP conjugates) and chemical modifications (LNA, 2′-O-methyl) from the earliest stages of preclinical development. Furthermore, the coordinated dysregulation of multiple ncRNAs in disease contexts suggests that single-target approaches may be limited by network redundancy, supporting the exploration of multi-target combination strategies.

In summary, this review positions ncRNAs as integral regulators of the signaling networks driving vascular calcification. The pathway-based classification, the identification of context-dependent duality in a subset of ncRNAs, and the delineation of mechanistic heterogeneity collectively provide a theoretical foundation for precision diagnosis and mechanism-based multi-target intervention.

## Figures and Tables

**Table 1:** miRNAs as potential biomarkers.

miRNA	Disease Context	Expression Change	Clinical Significance/Association
miR-106a-5p	CKD, ESRD, dialysis-related vascular calcification	Downregulated	Negatively correlated with calcification severity and serum PTH, osteocalcin
miR-223-3p, miR-93-5p		Downregulated	Expression recovers after renal transplantation
miR-211-5p		Gradually decreases with calcification progression	Potential biomarker for early detection of uremic vascular calcification
miR-31-5p		Low expression	Independent risk factor
miR-125b		Downregulated	Synergizes with osteoprotegerin to predict calcification risk
miR-143-5p		Reduced circulating level	Overexpression inhibits calcification
miR-145, miR-486		Downregulated in both calcified arteries and serum	Biomarkers
miR-126-3p, miR-145-5p	Arterial calcification	Downregulated	miR-126-3p predicts major adverse cardiovascular events (MACE)
miR-17-5p, miR-29a-3p	CAVD	Downregulated in serum	Consistent with changes in valve tissue
miR-223		Low baseline plasma expression	Associated with increased risk of adverse events after TAVI
miR-210		Upregulated	Independently associated with increased mortality
miR-92a		High expression	Positively correlated with transvalvular pressure gradient

**Table 2:** Context-dependent miRNAs (functional duality).

miRNA	Disease Context/Cell Type	Target Gene(s)	Functional Direction	Mechanistic Description
miR-21-5p	CAVD	TGFBI	Promotes calcification	Targets TGFBI, promotes VIC osteogenic differentiation
	CKD vascular calcification	Crim1	Promotes calcification	Delivered via adventitial fibroblast exosomes, targets Crim1 to promote VSMC osteogenesis
	Diabetic vascular calcification	Tpm1	Promotes calcification	Targets Tpm1; Danlian–Tongmai formula + intermittent fasting inhibits this axis
miR-155-5p	Normal (basal) conditions	RCN2	Inhibits calcification	Targets RCN2 to exert anti-calcification effect
	High-phosphate environment (CKD)	Negatively regulated by RCN2/STAT3 axis	Protective effect weakened	Forms RCN2/STAT3/miR-155-5p vicious feedback loop, weakening protection
	Uremic toxin (IS) induction	MGP	Promotes calcification	Upregulated via ROS/NF-κB, targets MGP
	Human peripheral arterial calcification lesions	CD73, Smad3	Promotes calcification	Upregulated, promotes VSMC osteogenesis by inhibiting CD73/Smad3
miR-145-5p	Vascular calcification (VSMC)	Smad3	Inhibits calcification	Directly binds and inhibits Smad3, prevents VSMC osteogenic differentiation
	CAVD	ZEB2	Promotes calcification	Enriched in EVs, targets ZEB2 to upregulate ALPL, promotes VIC osteogenesis

**Table 3:** lncRNAs regulating vascular calcification.

Function	lncRNA	Primary Mechanism	Target Molecule/Pathway	Disease Model
Inhibit calcification	HOTAIR	① ceRNA ② Epigenetic regulation	① miR-126 → Klotho → SIRT1 → inhibit Wnt/β-catenin; ② E2F1 → epigenetic silencing of KLF16 → derepress Klotho → inhibit Wnt/β-catenin	High-phosphate-induced VSMC
	GAS5	ceRNA	Sponges miR-26b-5p → upregulates PTEN	High-phosphate-induced VSMC
	SNHG29	ceRNA	Sponges miR-200b-3p → activates α-Klotho/FGFR1/FGF23 axis	High-phosphate-induced VSMC
	OIP5-AS1	ceRNA	Sponges miR-137 → upregulates TWIST1	CAVD (VICs)
	FGD5-AS1	ceRNA	Sponges miR-497-5p → upregulates BIRC5	CAVD
	SNHG1	Protein binding, mRNA stabilization	Stabilizes Bhlhe40 mRNA → inhibits Atg10 expression → inhibits autophagy	High-glucose-induced VSMC
	Lrrc75a-as1	Inhibits osteogenic genes	Inhibits Runx2, Msx2, BMP2 expression	High-phosphate-induced VSMC
	lncRNA-EPS	Inhibits inflammation + blocks Wnt/β-catenin	Inhibits inflammatory response, blocks Wnt signaling	Diabetes-associated VC
Promote calcification	NEAT1	ceRNA	miR-214-3p → upregulates PTEN → inhibits PI3K/AKT/mTOR	CAVD
	TUG1	ceRNA	miR-204-5p → upregulates Runx2	CAVD
	MALAT1	ceRNA	miR-30c → upregulates Runx2	VSMC & mesenchymal stem cells
	Prrc2c	ceRNA	miR-145-5p → upregulates Smad3	High-phosphate-induced VSMC
	SENCR	ceRNA	miR-4731-5p → activates PERK/ATF4 → upregulates Runx2	High-phosphate-induced VSMC
	NEAT1	Binds EZH2 (epigenetic regulation)	Binds EZH2 → inhibits transcription of P16/P21/TIMP3 → promotes proliferation/osteogenesis	Atherosclerosis
	SNHG3	Binds EZH2 (epigenetic regulation)	Interacts with EZH2 → removes H3K27me3 at BMP2 promoter → upregulates BMP2	CAVD
	MEG3	Activates signaling pathway	Activates Wnt/β-catenin pathway	CAVD
	lnc-COL6A1-6	Inhibits autophagy	Elevates autophagic activity, promotes osteogenic differentiation	CAVD
	AFAP1-AS1	Regulates inflammatory microenvironment	Promotes M1 macrophage polarization, inhibits M2 polarization	CAVD
	FAS-AS1	Regulates oxidative stress and inflammation	Elevates ROS, promotes TNF-α, IL-6	ESRD vascular calcification
	lincRNA-p21	Intercellular communication (small EV-mediated)	Promotes VSMC osteogenic transdifferentiation, apoptosis	High-phosphate-induced VSMC (CKD)
	ANRIL	Drives phenotypic transformation	Drives VSMC transition to osteochondrogenic-like state	Arterial calcification (9p21.3 risk locus)
Context-dependent	H19	Multiple pro-calcific mechanisms	① CKD: sponges miR-138 → activates NF-κB; ② Diabetes: inhibits BI-1/OPA1; ③ High phosphate: promoter CpG hypomethylation → activates Erk1/2	CKD, diabetes, high phosphate

Note: Arrows indicate regulatory relationships.

**Table 4:** circRNAs regulating vascular calcification.

Function	circRNA	Mechanism Category	Specific Mechanistic Description	Disease Model
Inhibit calcification	circSmoc1-2	Sponging (ceRNA)	Sponges miR-874-3p → upregulates Adam19	VSMC calcification
	circZBTB44	Peptide encoding (m^6^A modification)	Translates ZBTB44-342aa peptide → binds IGF2BP3 to stabilize CAMK1 → inhibits mitochondrial damage and cGAS-STING	CAVD
	circHIPK3	m^6^A modification + mRNA stabilization	DDX5/METTL3-mediated m^6^A modification → recruits IGF2BP2 to stabilize Krm1 mRNA → enhances inhibition of Wnt/β-catenin	CAVD
	circSMAD3	Molecular scaffold	Scaffold promotes interaction between hnRNPA1 and WDR76 → ubiquitination and degradation of hnRNPA1 → activates p53γ → inhibits VSMC phenotypic switching	Vascular injury/atherosclerosis
Promote calcification	circ-GALK2	Sponging (ceRNA)	Sponges miR-134-3p → upregulates CD36	VSMC calcification
	circTOP1	Regulates protein expression	Targets and regulates PTBP1 → promotes VSMC osteogenic phenotype transition	Coronary artery calcification
	circ_0008362	Sponging + direct protein binding	① Sponges miR-1251-5p to upregulate Runx2; ② Directly interacts with Runx2 protein	Diabetic vascular calcification
	circARHGAP10	Sponging (ceRNA)	Sponges miR-335-3p → upregulates RUNX2	CAVD
	circ-CCND1	Sponging	Sponges miR-138-5p → upregulates CCND1 → activates CCND1/P53/P21 pathway	CAVD
	circRIC3	Sponging	Sponges miR-204-5p → upregulates DPP4	CAVD

Note: Arrows indicate regulatory relationships.

**Table 5:** Priority ncRNAs with therapeutic and diagnostic potential.

ncRNA	Pathological Context	Key Mechanism (with Functional Direction)	Therapeutic/Diagnostic Potential
miR-21-5p	CAVD [12]; CKD [13]; diabetic VC [14]	Pro-calcific: targets TGFBI, Crim1, or Tpm1 depending on context	Target for natural product inhibition [14]
miR-133a	VSMC calcification [10]	Anti-calcific: downregulates Runx2 and osteocalcin	Classic anti-calcific miRNA; circulating levels predict LVH reversibility after AVR [111]
miR-204-5p	VSMC calcification [27,30,31]; CAVD [35]	Anti-calcific: targets Runx2 (VSMC) or Runx2/Osx (VIC)	Replacement therapy (human/animal/in vitro validated)
miR-223-3p	Medial and atherosclerotic calcification [49]	Anti-calcific: targets STAT3, blocks IL-6/STAT3; dual protection	Biomarker [20]; replacement therapy
circ_0008362	Diabetic vascular calcification [105]	Pro-calcific: sponges miR-1251-5p + directly binds Runx2	Diagnostic biomarker; therapeutic target
NEAT1	CAVD [92]; atherosclerosis [16]	Pro-calcific: ceRNA sponging miR-214-3p [92]; binds EZH2 [16]	ASO target (human/animal validated)
H19	CKD [47]; diabetes [101]; high phosphate [102]	Context-dependent pro-calcific: NF-κB (CKD); BI-1/OPA1 (diabetes); Erk1/2 (high Pi)	Context-specific target; requires selective delivery
circZBTB44	CAVD [18]	Anti-calcific: encodes peptide → binds IGF2BP3 → stabilizes CAMK1 → inhibits cGAS-STING	Peptide-based therapy (human/animal validated)

Note: Arrows indicate regulatory relationships.

## Data Availability

No new data were created or analyzed in this study. Data sharing is not applicable.

## Supplementary Materials

The following supporting information can be downloaded at: https://www.mdpi.com/article/10.3390/cells15151337/s1, Table S1. Comprehensive summary of miRNAs regulating vascular and valvular calcification.

## Abbreviations

The following abbreviations are used in this manuscript:

BMP	bone morphogenetic protein
CAVD	calcific aortic valve disease
ceRNA	competing endogenous RNA
circRNA	circular RNA
CKD	chronic kidney disease
CTGF	connective tissue growth factor
ER	endoplasmic reticulum
lncRNA	long non-coding RNA
MAPK	mitogen-activated protein kinase
miRNA	microRNA
NF-κB	nuclear factor kappa-light-chain-enhancer of activated B cells
OPG	osteoprotegerin
PI3K	phosphoinositide 3-kinase
RANKL	receptor activator of nuclear factor kappa-B ligand
Runx2	runt-related transcription factor 2
Smad	suppressor of mothers against decapentaplegic
STAT3	signal transducer and activator of transcription 3
TGF-β	transforming growth factor beta
VC	vascular calcification
VIC	valvular interstitial cell
VSMC	vascular smooth muscle cell
Wnt	wingless-type MMTV integration site family
Dkk2	Dickkopf WNT Signaling Pathway Inhibitor 2
SULF1	Sulfatase 1
GSK-3β	Glycogen Synthase Kinase 3 Beta
CK1	Casein Kinase 1
Axin	Axis Inhibition Protein
APC	Adenomatous Polyposis Coli
TGF-βRII	Transforming Growth Factor Beta Receptor II
Osterix/SP7	Osterix (Zinc Finger Transcription Factor SP7)
SOX9	SRY-box Transcription Factor 9
CDH11	Cadherin-11
TLR3	Toll-like Receptor 3
IL-6	Interleukin 6
SOCS1	Suppressor of Cytokine Signaling 1
NLRP3	NLR Family Pyrin Domain Containing 3
AKT	AKT serine/threonine kinase
VWF	Von Willebrand Factor
IGF	Insulin-like Growth Factor
VEGF	Vascular Endothelial Growth Factor
IGF-1	Insulin-like Growth Factor 1
ERK	Extracellular Signal-Regulated Kinase
p38	p38 Mitogen-Activated Protein Kinase
RAF	Rapidly Accelerated Fibrosarcoma Kinase
MEK	MAPK/ERK Kinase
