# Supplementary material for: Non-Coding RNAs in Vascular Calcification: Regulatory Networks, Functional Diversity, and Therapeutic Insights
Source: Cells. 2026 Jul 25;15(15):1337. doi: 10.3390/cells15151337 (PMC13464055; doi:10.3390/cells15151337)
Supplement: Supplementary file 1 [file cells-15-01337-s001.zip › cells-4432841-supplementary.pdf]

Table S1. Comprehensive summary of miRNAs regulating vascular and valvular calcification

Signaling pathway	miRNA	Disease context / cell type	Target gene(s)	Functional direction	Mechanistic description	Evidence level
Wnt/β-catenin	miR-302d-5p	VSMC calcification	Wnt3	Inhibits	Endothelial cell-derived exosomal miR-302d-5p targets Wnt3, blocks canonical Wnt/β-catenin signaling, alleviates VSMC calcification and senescence	in vitro
	miR-182-5p	CAVD (VICs)	Dkk2	Promotes	Targets and inhibits Dkk2, relieves negative regulation of Wnt/β-catenin by Krm1-Dkk2 complex, promotes VIC osteogenic differentiation	in vitro
	miR-378a-3p	VSMC calcification	SULF1	Inhibits	Targets and inhibits SULF1, blocks Wnt/β-catenin signaling; downregulated in CKD serum	human; in vitro
	miR-26a	VSMC calcification	CTGF	Inhibits	Targets CTGF (downstream effector of Wnt/β-catenin), regulates OPG/RANKL signaling	in vitro
	miR-138-5p	CAVD (VICs)	RUNX2	Inhibits	Directly targets RUNX2, blocks Wnt/β-catenin signaling, inhibits osteogenic differentiation and calcium deposition	in vitro
	miR-204-5p	VSMC calcification	RUNX2	Inhibits	Delivered via exosomes from AT2 agonist-stimulated macrophages, targets RUNX2, blocks Wnt/β-catenin	animal; in vitro
	miR-433-3p	Type 2 diabetes (serum)	Wnt/β-catenin, RANKL/RANK/OPG	Inhibits	Downregulated in serum, relieves inhibition of Wnt/β-catenin and RANKL/RANK/OPG, indirectly promotes calcification	human
BMP/Smad/Runx2	miR-211	VSMC calcification	Runx2	Inhibits	Downregulated in high-phosphate-induced calcification; direct inhibition of Runx2 is weakened, promoting calcification	in vitro
	miR-204	VSMC calcific	Runx2	Inhibits	Downregulated due to DNMT3a-mediated promoter	animal; in vitro

Signaling pathway	miRNA	Disease context / cell type	Target gene(s)	Functional direction	Mechanistic description	Evidence level
		ation			hypermethylation in high phosphate; overexpression reverses calcification	
	miR-205	VSMC calcification	Runx2, Smad1	Inhibits	Negatively regulates osteogenic-like differentiation by targeting Runx2 and Smad1	in vitro
	miR-133a	VSMC calcification	Runx2	Inhibits	Downregulated in BMP2-induced model; overexpression reduces calcification by downregulating Runx2 and osteocalcin; circulating levels predict LVH reversibility after AVR	human; in vitro
	miR-30b/c	VSMC calcification	Runx2	Inhibits	Directly bind Runx2 mRNA; levels reduced in calcified human coronary arteries	human; in vitro
	miR-335	VSMC calcification	SP1 (→RUNX2)	Inhibits	BMSC-derived exosomal miR-335 targets SP1 to downregulate RUNX2, antagonizes calcification	in vitro
	miR-3960	VSMC calcification	Hoxa2	Promotes	Upregulated during osteogenic transdifferentiation; targets Hoxa2, increases Runx2; forms positive feedback loop with Runx2	in vitro
	miR-2861	VSMC calcification	HDAC5	Promotes	Upregulated during osteogenic transdifferentiation; targets HDAC5, increases Runx2; forms positive feedback loop with Runx2	in vitro
	miR-17-5p	VSMC calcification	TGF-βRII	Inhibits	Macrophage-derived EVs deliver miR-17-5p, targets TGF-βRII, attenuates TGF-β1-induced osteogenic differentiation	in vitro
	miR-302b	VSMC calcification	BMP-2/Runx2/Osterix	Inhibits	Downregulated in chronic renal failure model; overexpression inhibits BMP-2/Runx2/Osterix signaling, alleviates calcification	animal
	miR-103a-	CAVD (VICs)	BMP4/R	Inhibits	Downregulated in plasma EVs from aortic stenosis patients;	human; in vitro

Signaling pathway	miRNA	Disease context / cell type	Target gene(s)	Functional direction	Mechanistic description	Evidence level
	3p		UNX2		inhibits VIC osteogenic differentiation via BMP4/RUNX2	
	miR-455-3p	CAVD (VICs)	BMP4/RUNX2	Promotes	Upregulated in plasma EVs; inhibition alleviates VIC osteogenic differentiation via BMP4/RUNX2	human; in vitro
	miR-26b	VSMC calcification	Smad1	Inhibits	Targets Smad1, blocks fibroblast-derived BMP4-mediated pro-calcific communication; loss drives aortic microcalcification	animal; in vitro
	miR-145-5p	VSMC calcification	Smad3	Inhibits	Directly binds and inhibits Smad3, prevents VSMC osteogenic differentiation	in vitro
	miR-449c-5p	CAVD (VICs)	Smad4	Inhibits	Downregulated in calcified valves; targets Smad4, blocks TGF-β/Smad, suppresses VIC osteogenic differentiation	human; in vitro
	miR-125b	VSMC calcification	SP7 (Osterix)	Inhibits	Negatively regulates osteogenic-like differentiation and calcification by targeting SP7	in vitro
	miR-638	CAVD (VICs)	SP7 (Osterix)	Inhibits	Upregulated in calcified valves (compensatory); exerts anti-osteogenic effect by targeting SP7	human; in vitro
	miR-101-3p	CAVD (VICs)	CDH11, SOX9	Promotes	Upregulated in calcified valves; targets CDH11 and SOX9, promotes VIC osteogenic differentiation	human; in vitro
NF-κB	miR-138	VSMC calcification	TLR3	Inhibits	Binds TLR3 mRNA, blocks NF-κB activation, inhibits vascular calcification	in vitro
	miR-155-5p	VSMC calcification	MGP	Promotes	Upregulated via ROS/NF-κB by uremic toxin IS; targets MGP, promotes calcification	in vitro
	miR-223-3p	VSMC calcification	STAT3	Inhibits	Blocks IL-6/STAT3 by targeting STAT3, prevents VSMC osteoblast-like transition; dual protection against medial and atherosclerotic calcification	animal; in vitro
	miR-	CAVD	STA	Promot	Upregulated in CAVD; targets	in vitro

Signaling pathway	miRNA	Disease context / cell type	Target gene(s)	Functional direction	Mechanistic description	Evidence level
	29b	(VICs)	T3	es	STAT3, downregulates SOCS1, activates NLRP3 inflammasome-mediated pyroptosis, promotes VIC osteogenic differentiation	
	miR-34a	VSMC calcification (aging)	SIRT6	Promotes	Targets SIRT6, weakens Keap1/NRF2 antioxidant defense, promotes aging-induced medial calcification; liraglutide reverses by downregulating miR-34a	animal; in vitro
MAPK / PI3K/ AKT	miR-195	CAVD (VICs)	VWF	Inhibits	Downregulated in CAVD; targets VWF, blocks p38-MAPK, alleviates VIC osteogenic differentiation	in vitro
	miR-30e	VSMC calcification	IGF2	Inhibits	Highly expressed in normal vessels; targets IGF2, inhibits osteogenic differentiation; downregulated in aging/atherosclerosis	animal; in vitro
	miR-29a-3p	VSMC calcification	Vegfa	Inhibits	Targets Vegfa, alleviates high-phosphate-induced VSMC calcification	in vitro
	miR-16-5p	VSMC calcification	VEGFA-VEGFR2	Inhibits	Coordinated deficiency in CKD sEVs relieves inhibition of VEGFA-VEGFR2, drives VSMC osteogenic transformation	human; animal; in vitro
	miR-17-5p	VSMC calcification	VEGFA-VEGFR2	Inhibits	Coordinated deficiency in CKD sEVs; predictive value for calcification	human; animal; in vitro
	miR-20a-5p	VSMC calcification	VEGFA-VEGFR2	Inhibits	Coordinated deficiency in CKD sEVs; predictive value for calcification	human; animal; in vitro
	miR-106b-5p	VSMC calcification	VEGFA-VEGFR2	Inhibits	Coordinated deficiency in CKD sEVs; predictive value for calcification	human; animal; in vitro
	miR-670-3p	VSMC calcification	IGF-1	Promotes	Enriched in high-phosphate-induced endothelial exosomes; targets IGF-1, promotes VSMC osteogenic differentiation; plasma levels correlate with CAC severity	human; in vitro

Signaling pathway	miRNA	Disease context / cell type	Target gene(s)	Functional direction	Mechanistic description	Evidence level
	miR-32	VSMC calcification (T2D)	FoxO/MAPK	Promotes	Elevated in T2D plasma; promotes endothelial ferroptosis; BMSC-EVs inhibit miR-32-mediated ferroptosis to alleviate calcification	human; animal; in vitro
	miR-22	CAVD (VICs)	CAB39	Promotes	Upregulated in calcified valves; targets CAB39, weakens CAB39-LKB1-STRAD complex, inhibits AMPK-mTOR, promotes VIC osteogenic differentiation	human; in vitro
ER stress / Autophagy	miR-199a-5p	CAVD (VICs)	ATF6, GRP78	Inhibits	Downregulated in CAVD; targets ATF6 and GRP78, blocks ER stress branch, suppresses VIC osteogenic differentiation	in vitro
	miR-4731-5p	VSMC calcification	PERK/ATF4	Inhibits	Downregulated in β-glycerophosphate-induced calcification; inhibits PERK/ATF4-mediated ER stress, alleviates osteogenic differentiation	in vitro
	miR-129	CAVD (VICs)	eIF2α/ATF4	Inhibits	Exosomal from intermittent hypoxia-stimulated VSMCs; cooperatively targets eIF2α/ATF4, attenuates VIC osteogenic differentiation	in vitro
	miR-342	CAVD (VICs)	eIF2α/ATF4	Inhibits	Exosomal from intermittent hypoxia-stimulated VSMCs; cooperatively targets eIF2α/ATF4, attenuates VIC osteogenic differentiation	in vitro
	miR-214	CAVD (VICs)	ATF4, Sp7	Inhibits	Downregulated in calcified valves; targets ATF4 and Sp7, suppresses VIC osteogenic differentiation; knockout exacerbates calcification	animal; in vitro
	miR-320a-3p	VSMC calcification	PDCD4	Inhibits	Cold exposure-induced plasma exosomes enriched; activates AMPK/mTOR autophagy by targeting PDCD4, suppresses VSMC osteogenic differentiation	animal; in vitro
	miR-181c-5p	VSMC calcification (CKD)	SIRT1	Promotes	Upregulated in CKD; targets SIRT1, inhibits Pink1/Parkin-mediated mitophagy, promotes ferroptosis, drives VSMC osteogenic	in vitro

**Signaling pathway**	**miRNA**	**Disease context / cell type**	**Target gene(s)**	**Functional direction**	**Mechanistic description**	**Evidence level**
					differentiation	
Notch	miR-29a-5p	CAVD (VICs)	GSAP	Inhibits	Downregulated by PTH; relieving GSAP inhibition activates γ-secretase/Notch1, inducing EndMT and promoting valve calcification	animal; in vitro
	miR-34b	VSMC calcification	Notch1	Inhibits	Targets Notch1, inhibits VSMC osteoblast-like differentiation; suppressed at cellular, animal, and uremic patient levels	human; animal; in vitro
ECM / Calcification inhibitors	miR-29b-3p	VSMC calcification	MMP2	Inhibits	Directly targets MMP2, suppresses vascular calcification	in vitro
	miR-874-3p	VSMC calcification	Adam19	Promotes	Targets Adam19 (anti-calcification gene), promotes VSMC calcification	animal; in vitro
	miR-143-3p	VSMC calcification	MGP	Promotes	Targets MGP; sponged by MALAT1 in macrophage exosomes under high glucose, relieving MGP inhibition	animal; in vitro
	miR-181b	CAVD (VICs)	TIMP3	Promotes	Upregulated in valvular endothelial cells under oscillatory shear; targets TIMP3, enhances MMP activity, promotes ECM degradation and CAVD progression	in vitro
